# How consistent is the interpretation of renal scarring in pediatric patients using technetium-99m dimercaptosuccinic acid scintigraphy

**DOI:** 10.1007/s00247-025-06379-z

**Published:** 2025-08-26

**Authors:** Mansura Naznine, Muhammad E. H. Chowdhury, Abdus Salam, Abdusamea Shabani, Karuppiah Kumaresan, Nadezhda Komarova, Amitabh Arya, Takahiro Hosokawa, Khalsa Zahran Al-Nabhani, Tariq O. Abbas

**Affiliations:** 1 Department of Electrical Engineering, Qatar University, Doha 2713, Qatar; 2 Department of Computer Science & Engineering, Rajshahi University of Engineering & Technology, Rajshahi, Bangladesh; 3 Department of Electrical and Computer Engineering, Rajshahi University of Engineering & Technology, Rajshahi, Bangladesh; 4 Radiology Department, Sidra Medicine, Doha, Qatar; 5 KK Nuclear Scans, Hyderabad, India; 6 Federal Clinical Research Centre of Russian Federal Medical-Biological Agency, Department of Nuclear Medicine, Moscow, Russian Federation; 7 Department of Nuclear Medicine, SGPGIMS, Lucknow, India; 8 Saitama Children’s Medical Center, Japan, Japan; 9 Head of Nuclear Medicine Department & Molecular Imaging Centre, Royal Hospital, Muscat, Oman; 10 Urology Division, Surgery Department, Sidra Medicine, Doha, 26999 Qatar; 11 College of Medicine, Qatar University, Doha, Qatar

**Keywords:** Cohen’s kappa, Intra-observer agreement, Inter-observer agreement, Kendall’s tau-b, Pediatric patients, Technetium-99m dimercaptosuccinic acid

## Abstract

**Background:**

Technetium-99m dimercaptosuccinic acid (DMSA) scintigraphy plays a critical role in pediatric imaging for detecting renal cortical scarring, which is essential for diagnosing and managing kidney damage in children. However, variability in observer interpretation poses challenges, potentially impacting clinical decision-making and outcomes.

**Objective:**

This study aims to assess intra- and inter-observer agreement in interpreting DMSA scans for detecting renal cortical scarring in pediatric patients, focusing on the presence, location, and percentage of kidney involvement.

**Materials and methods:**

This prospective study analyzed 220 pediatric patients with suspected renal scarring. Four experienced radiologists independently reviewed DMSA scans on two separate occasions, 3–4 weeks apart, using standardized assessment criteria. Intra-observer agreement was measured using Cohen’s kappa, while inter-observer agreement was assessed using pairwise Cohen’s kappa for categorical evaluations and Kendall’s tau-b for the percentage of kidney involvement.

**Results:**

Strong intra-observer agreement was observed across all four radiologists, with Cohen’s kappa values for renal scarring stages ranging from 0.704 to 0.955. Observer-4 consistently showed the highest agreement across all metrics. Inter-observer agreement varied substantially depending on observer pairs. Pairs excluding Observer-2 demonstrated moderate to substantial agreement (kappa up to 0.8268 and Kendall’s tau-b up to 0.7192), while pairs involving Observer-2 showed poor to slight agreement. Variability was particularly notable in assessing scarring severity and defect localization.

**Conclusion:**

While intra-observer consistency in interpreting DMSA scans is high, inter-observer variability remains a concern, especially in evaluating the severity and location of renal scarring. These findings underscore the need for standardized protocols and targeted training to enhance diagnostic accuracy. Moreover, the development of validated datasets could support the advancement of machine learning models for automated, precise detection of renal scarring, ultimately improving diagnostic reliability and patient outcomes.

**Graphical Abstract:**

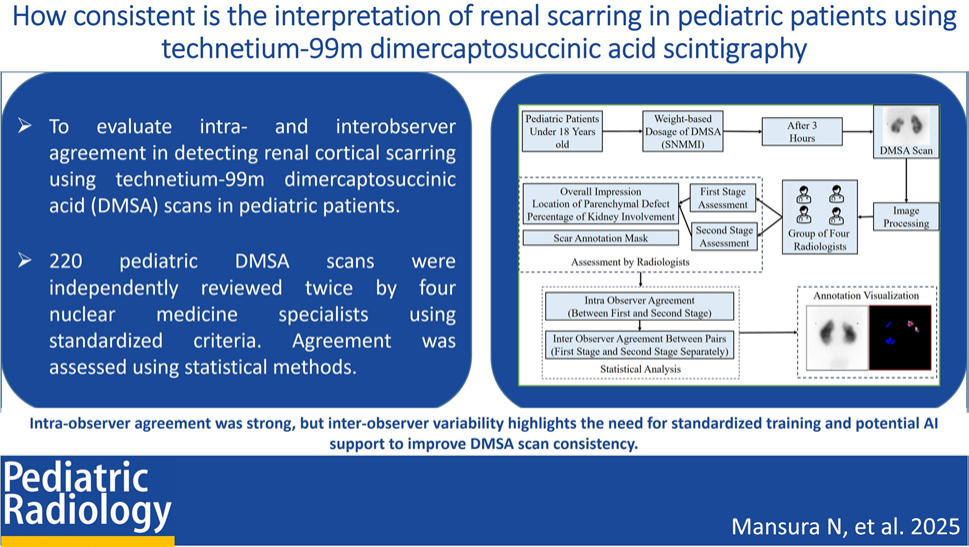

**Supplementary Information:**

The online version contains supplementary material available at 10.1007/s00247-025-06379-z.

## Introduction

Urinary tract infections (UTIs) affect 7.8% of females and 1.7% of boys by age seven. UTIs may involve the upper urinary tract (pyelonephritis) or lower tract (cystitis), and if left untreated, can lead to renal scarring and long-term complications such as hypertension and chronic kidney disease [1]. Early detection of newborn UTIs can be challenging due to nonspecific signs including fever, agitation, and poor feeding, while recurrent UTIs increase the risk of lasting renal damage [2]. These challenges highlight the need for accurate and timely assessment of renal involvement to guide long-term management [3, 4].

Technetium-99m dimercaptosuccinic acid (DMSA) scintigraphy is widely recognized as the most sensitive imaging modality for detecting renal cortical defects in children and is primarily employed to assess renal parenchymal involvement and long-term sequelae such as scarring several months after a urinary tract infection (UTI), especially following episodes of acute pyelonephritis [5]. Acute pyelonephritis involves infection and inflammation of the renal parenchyma and can result in irreversible damage to the kidney if not effectively treated [6]. While urine culture remains the gold standard for diagnosing UTI during the acute phase, DMSA scintigraphy serves a different but complementary role in assessing whether and to what extent the kidneys have been affected by the infection. In clinical settings, a top-down approach is adopted, where DMSA is performed during the acute phase of UTI to detect renal parenchymal involvement before proceeding to more invasive investigations. By providing high-resolution anatomical images and highlighting areas of reduced or absent cortical uptake, DMSA scans allow clinicians to determine the extent and distribution of renal defects including scars [7, 8]. This information is vital for risk stratification, long-term monitoring, and intervention planning in pediatric patients who are vulnerable to progressive renal damage and its associated complications.

Despite its diagnostic value, DMSA scan interpretation is inherently subjective and influenced by the reader’s experience and skill. Additional variability may arise from the radiopharmaceutical’s quality, imaging system precision, and criteria used to define scarring or presence of renal failure. Previous studies have shown that such variability can result in inconsistent diagnoses, ultimately impacting treatment decisions and long-term outcomes in children with UTIs [9]. To assess overall reliability of DMSA scintigraphy, it is important to quantify a single observer’s consistency of scan interpretation over time, but also assess the extent of agreement between different observers. Low agreement indicates the need for standardization and training to reduce subjective variance, while high agreement between and among observers indicates good diagnostic technique (which can be further improved by standardized reading processes, automated or semi-automatic image processing, and regular inter-observer calibration) [9, 10]. The primary objective of this study was to assess observer reliability in interpreting DMSA scans for detecting renal scarring in pediatric patients. Specifically, the study aimed to evaluate intra-observer and inter-observer agreement, with a focus on determining consistency of interpretation by individual observers over time (intra-observer agreement) and the level of agreement between different observers (inter-observer agreement). The hypothesis was that low agreement would highlight the need for improved standardization and training, which could be addressed through regular calibration and the use of automated or semi-automated image processing techniques.

## Materials and methods

To ensure a rigorous and comprehensive evaluation of renal cortical defects in pediatric patients, this retrospective cohort study was designed to assess both inter-observer and intra-observer agreement using DMSA scan.

### Collection of patient information

**Fig. 1:**
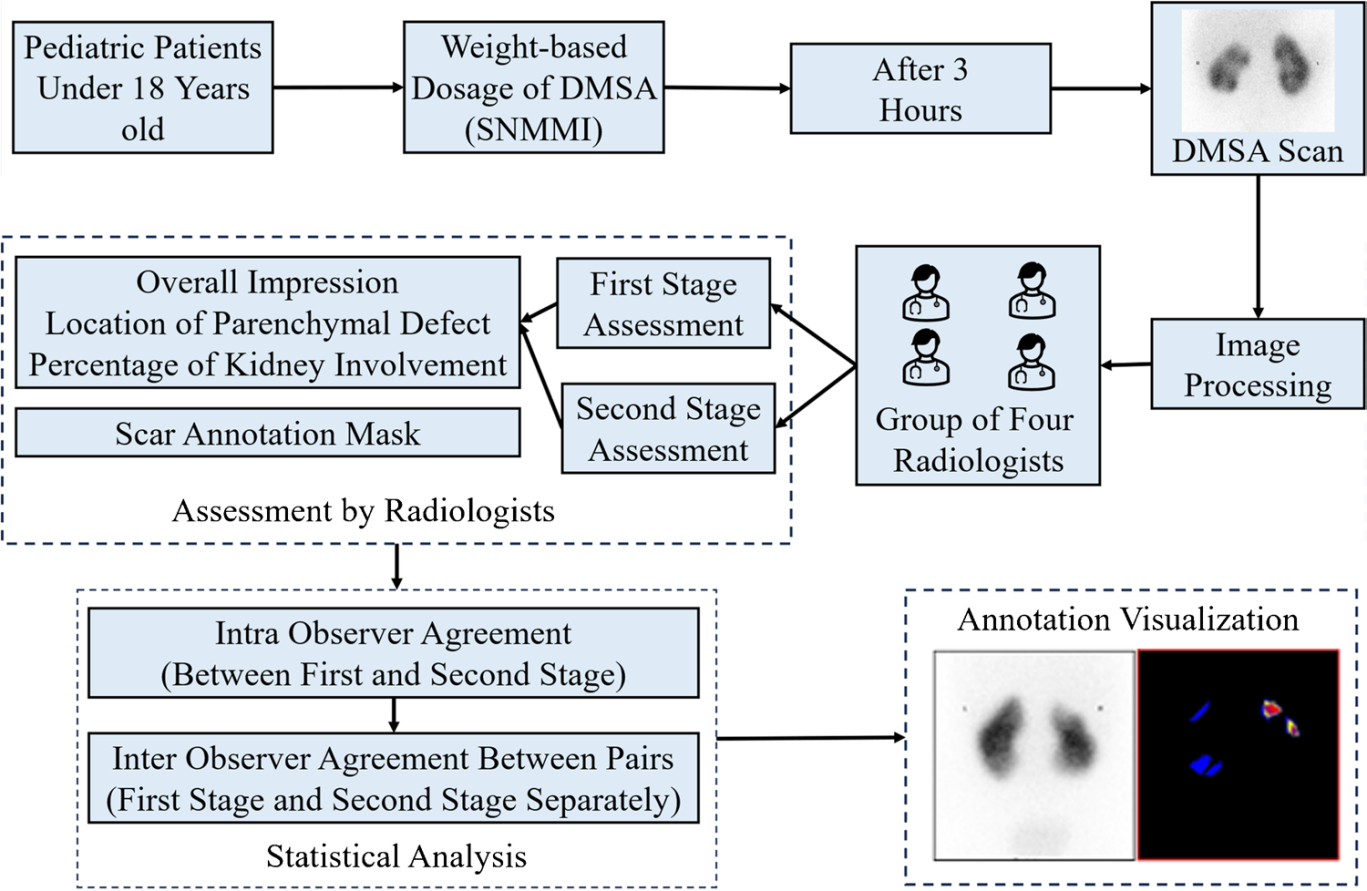
Methodology used in this study. *DMSA* technetium-99m dimercaptosuccinic acid, S*NMMI* Society of Nuclear Medicine and Molecular Imaging

The study included patient information from a cohort of 693 individuals who underwent DMSA scans, from which 220 patients were selected for evaluation. The study cohort included pediatric patients defined as individuals aged <18 years. No patients who had attained the age of 18 years were included, in accordance with both the clinical practice at our center and the dataset criteria. Each patient underwent imaging of both kidneys, and selection criteria included high-quality scans with complete visualization of renal cortices and representative sections of the entire kidney. The median age of the selected patients was 50.0 months (interquartile range [IQR]: 19.75–111.25 months), and the cohort comprised 120 males and 100 females. DMSA scans in this study were primarily performed for the evaluation of urinary tract infections (UTIs)—to detect acute pyelonephritis or renal scarring—and for the investigation of congenital anomalies of the kidney and urinary tract. Based on clinical interpretations, 287 kidneys were classified as normal (No Scar), and 153 kidneys were identified as having renal scarring (Scar). Data were compiled from Cerner PowerChart, integrating previous diagnostic imaging procedures, physician documentation notes, and microbiology results retrieved from laboratory records. Figure 1 demonstrates the overall methodology used in this study.

### Protocol for technetium-99m dimercaptosuccinic acid scanning

The technetium-99m dimercaptosuccinic acid (DMSA) scan protocol adhered to established guidelines to ensure consistent and accurate assessment of renal cortical defects. Each patient received a weight-based DMSA dose calculated using the Society of Nuclear Medicine and Molecular Imaging (SNMMI) pediatric dosing program. The administered dose ranged from a minimum of 18.5 MBq (0.5 mCi) to a maximum of 110 MBq (3.0 mCi), ensuring optimal image quality while maintaining compliance with radiation safety standards. Imaging was performed approximately 3 h after injection using a Siemens Symbia Intevo Bold gamma camera (Siemens Healthineers, Erlangen, Germany) equipped with high-resolution collimators. Static anterior and posterior images were obtained in the supine position, followed by single-photon emission computed tomography (SPECT) acquisition using standard clinical protocols. Only posterior static images were used for observer evaluation. Routine quality control procedures, including gamma camera uniformity checks and dose calibrator assessments, were performed to maintain imaging accuracy. An example DMSA scan is shown in Supplementary Material 1.

### Interpretation of scans

Every DMSA scan was independently assessed on two separate occasions approximately 3–4 weeks apart by four nuclear medicine specialists with varying levels of expertise and experience. Each specialist had a minimum of 10 years of experience in pediatric renal imaging interpretation, and all routinely interpret DMSA scans as part of their clinical practice.

- Observer-1: 10 years of experience in pediatric nuclear medicine.
- Observer-2: 12 years of experience in pediatric nuclear medicine.
- Observer-3: 10 years of experience with a subspecialty focus in renal imaging.
- Observer-4: 11 years of experience as a senior consultant in pediatric nuclear medicine.

To promote consistency in evaluation, a brief calibration session was conducted prior to the study assessments to review and align on standardized interpretation criteria. Observers were blinded to all clinical information, including patient history, diagnosis, and treatment status. They were also blinded to each other’s assessments and to the original time point of scan acquisition. To minimize recall bias, the order of DMSA scan images was randomized prior to each review session. Observers independently reviewed the images without access to previous annotations or scoring. To minimize recall bias, the order of image presentation was randomized between the two reading sessions. Observers were unaware that a scan being reviewed at the second session may have been previously assessed by them, ensuring blinding to the time point of the scan. For each scan, the radiologists provided their assessment of whether scars were present or absent, identified the specific locations of any defects, and quantified the percentage of kidney involvement in these defects. The uniform criteria for interpreting DMSA scans are outlined as follows:

- Overall impression: Kidney scans were classified into two categories—Scar and No Scar.
- Location of parenchymal defect: Not Affected, Upper Pole, Mid-Zone, Lower Pole, or Multiple Zones if more than one area is involved.
- Percentage of kidney involvement: quantification of affected parenchyma using the following categories: 0%, <10%, 10–24%, 25–49%, 50–74%, and >74%.

In addition, each radiologist created binary manual annotation files delineating the kidney regions affected by scars using Make Sense AI (MakeSense.AI, Krakow, Poland) (https://www.makesense.ai/). This platform enables precise and standardized delineation of affected areas across all scans (an example file is shown in Supplementary Material 2). ROIs were manually annotated using the Make Sense AI platform, without the use of any AI-assisted or automated segmentation features. Each observer independently annotated the regions of interest. In cases where annotations differed, a majority vote among the three observers was used to determine the final annotation, which was designated as the ground truth for subsequent analysis. The manually annotated DMSA scans generated in this study not only supported the observer variability analysis but also served as a foundation for creating high-quality training data for potential AI-based interpretation tools. It also provides a valuable foundation for developing and validating AI-based interpretation tools.

### Observer agreement

Overall impression and location of parenchymal defect: Consistency and agreement among radiologists were evaluated through intra-observer and inter-observer analyses using Cohen’s kappa (κ) [11]. According to established guidelines, a kappa statistic of 0 indicates poor agreement, 0.01–0.20 slight agreement, 0.21–0.40 fair agreement, 0.41–0.60 moderate agreement, 0.61–0.80 substantial agreement, and 0.81–1.00 almost perfect agreement (based on guidelines proposed by Landis and Koch) [12].

Percentage of kidney involvement: The degree of concordance in the percentage of kidney involvement was analyzed using Kendall’s tau-b $({\tau }_{b})$ [13]. The value of Kendall’s tau-b ranges from −1 to 1, where values closer to 1 indicate strong positive concordance, values closer to −1 indicate strong negative concordance, and values near 0 indicate no concordance. Intra-observer agreement was assessed by calculating ${\tau }_{b}$ for each radiologist between the first and second observations. For inter-observer agreement, ${\tau }_{b}$ values were computed for each pair of observers providing a comprehensive evaluation of concordance between specific pairs.

## Results

### Intra-observer agreement

**Fig. 2:**
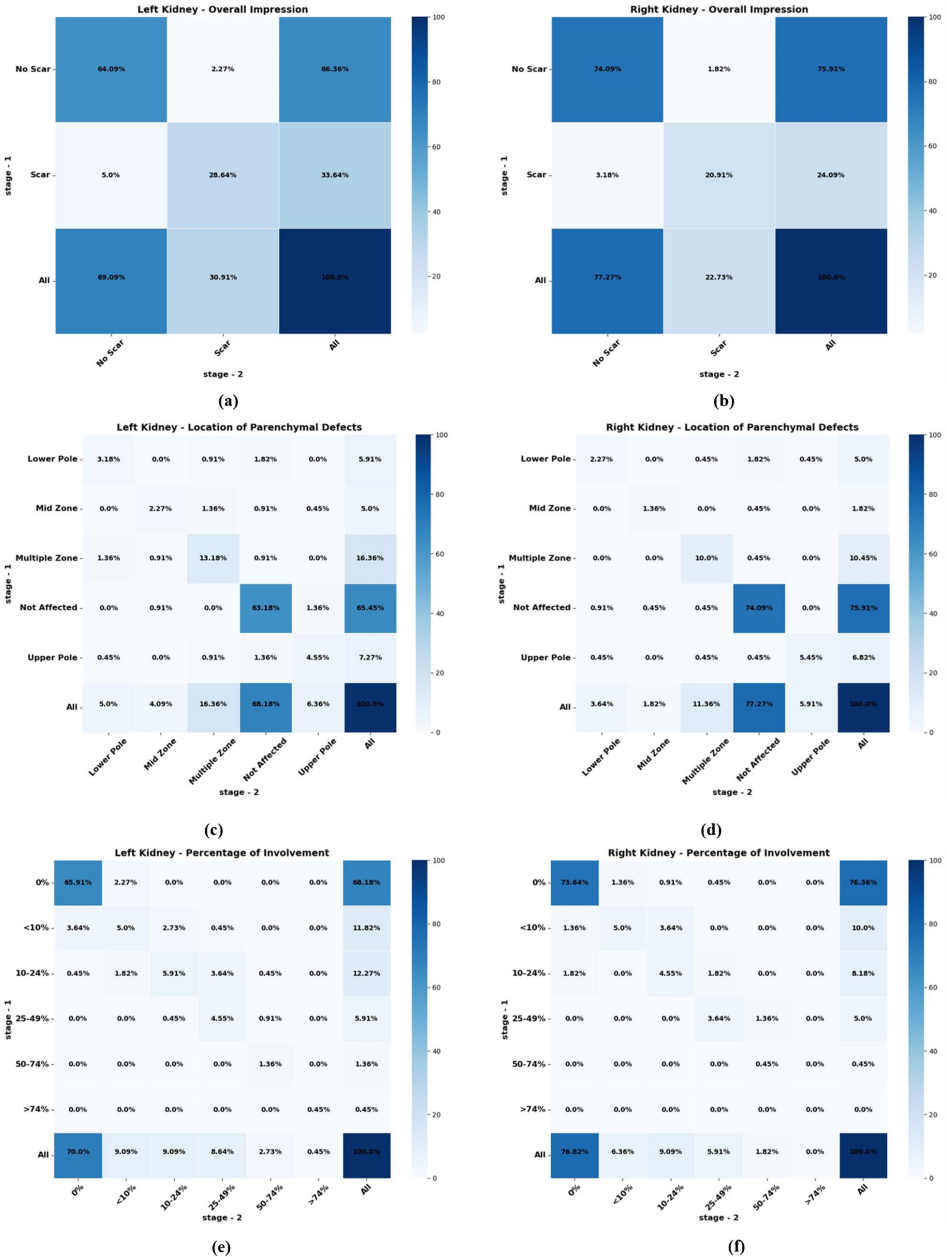
Heatmap visualization of intra-observer agreement for Observer-3. Cohen’s kappa cross-tabulations for overall impression: (**a**) left kidney, (**b**) right kidney. Cohen’s kappa cross-tabulations for location of parenchymal defect: (**c**) left kidney, (**d**) right kidney. Kendall’s tau-b cross-tabulations for percent involvement of (**e**) left kidney and (**f**) right kidney

Consistency and reliability of DMSA renal scan evaluations across four observers were critically examined using various parameters displayed in Table 1. Cohen’s kappa scores for renal scarring ranged from 0.704 to 0.95, indicating substantial to almost perfect agreement. Observer-4 achieved the highest agreement (0.91 for the left kidney, 0.95 for the right), while Observer-1 reported the lowest (0.703 left, 0.77 right). For parenchymal defect detection, Observer-2 had the lowest kappa values (0.504 left, 0.55 right), and Observer-4 had the highest (0.89 left, 0.91 right). Regarding the percentage of kidney involvement, Kendall’s tau-b values also varied across observers. Observer-4 demonstrated the highest consistency (0.87 left, 0.91 right), whereas Observer-2 showed the lowest agreement (0.43 left, 0.52 right). These metrics highlight both the reliability and the degree of variation in individual observer assessments. Figure 2 presents the heatmap visualization of intra-observer agreement for Observer-3. A detailed breakdown of intra-observer agreement results is provided in Supplementary Materials 3, 5, 7, and 8, which contain observer-specific result tables. Corresponding heatmaps and cross-tabulations illustrating Cohen’s kappa and Kendall’s tau-b statistics for each observer are shown in Supplementary Materials 4, 6, and 9. These visualizations further clarify the agreement patterns for overall impression, location of parenchymal defects, and percent involvement across the left and right kidneys.

**Table 1:** Intra-observer agreement for technetium-99m dimercaptosuccinic acid (DMSA) renal scintigraphy evaluations across four observers: Cohen’s kappa score for overall impression and location of parenchymal defect, Kendall’s tau-b coefficients for percentage of kidney involvement

Observer	Aspect of agreement	Kidney	Measure	Score
Observer-1	Overall impression	Left	Cohen’s kappa	0.703
		Right	Cohen’s kappa	0.77
	Location of parenchymal defect	Left	Cohen’s kappa	0.602
		Right	Cohen’s kappa	0.64
	Percentage of kidney involvement	Left	Kendall’s tau-b	0.62
		Right	Kendall’s tau-b	0.69
Observer-2	Overall impression	Left	Cohen’s kappa	0.704
		Right	Cohen’s kappa	0.74
	Location of parenchymal defect	Left	Cohen’s kappa	0.504
		Right	Cohen’s kappa	0.55
	Percentage of kidney involvement	Left	Kendall’s tau-b	0.43
		Right	Kendall’s tau-b	0.52
Observer-3	Overall impression	Left	Cohen’s kappa	0.83
		Right	Cohen’s kappa	0.86
	Location of parenchymal defect	Left	Cohen’s kappa	0.73
		Right	Cohen’s kappa	0.82
	Percentage of kidney involvement	Left	Kendall’s tau-b	0.73
		Right	Kendall’s tau-b	0.78
Observer-4	Overall impression	Left	Cohen’s kappa	0.91
		Right	Cohen’s kappa	0.95
	Location of parenchymal defect	Left	Cohen’s kappa	0.89
		Right	Cohen’s kappa	0.909
	Percentage of kidney involvement	Left	Kendall’s tau-b	0.86
		Right	Kendall’s tau-b	0.91

### Inter-observer agreement

Table 2 presents the inter-observer agreement metrics for DMSA scan evaluations, including Cohen’s kappa scores for scar presence (“Overall Impression”) and the location of parenchymal defects, as well as Kendall’s tau-b values for percentage of kidney involvement. Observer pairs involving Observer-2 (e.g., 1 vs. 2, 2 vs. 3, 2 vs. 4) consistently showed the lowest agreement across all evaluated parameters and stages. For the left kidney, kappa scores ranged from 0.0801 to 0.22, indicating poor to slight agreement, while corresponding Kendall’s tau-b values ranged from 0.12 to 0.303. In contrast, observer pairs that excluded Observer-2 (e.g., 1 vs. 3, 1 vs. 4, 3 vs. 4) demonstrated higher agreement. The 1 vs. 4 pair showed the highest consistency across all metrics, with kappa scores reaching substantial agreement (e.g., 0.64–0.82) and Kendall’s tau-b values between 0.57 and 0.71 for both kidneys. These results highlight substantial variability in agreement between observer pairs.

**Table 2:** Inter-observer agreement for Technetium-99m dimercaptosuccinic acid (DMSA) scans for observer pairs, Cohen*’*s kappa score for overall impression and location of parenchymal defects, Kendall’s tau-b for percentage of kidney involvement

Stage	Observer pair	Aspect of agreement	Measure	Score of left kidney	Score of right kidney
Stage-1	1 vs. 2	Overall impression	Cohen’s kappa	0.14	0.22
		Location of parenchymal defect	Cohen’s kappa	0.18	0.18
		Percentage of kidney involvement	Kendall’s tau-b	0.23	0.36
	1 vs. 3	Overall impression	Cohen’s kappa	0.52	0.62
		Location of parenchymal defect	Cohen’s kappa	0.46	0.49
		Percentage of kidney involvement	Kendall’s tau-b	0.46	0.53
	1 vs. 4	Overall impression	Cohen’s kappa	0.69	0.82
		Location of parenchymal defect	Cohen’s kappa	0.63	0.65
		Percentage of kidney involvement	Kendall’s tau-b	0.59	0.71
	2 vs. 3	Overall impression	Cohen’s kappa	0.12	0.14
		Location of parenchymal defect	Cohen’s kappa	0.13	0.13
		Percentage of kidney involvement	Kendall’s tau-b	0.12	0.034
	2 vs. 4	Overall impression	Cohen’s kappa	0.14	0.18
		Location of parenchymal defect	Cohen’s kappa	0.22	0.22
		Percentage of kidney involvement	Kendall’s tau-b	0.206	0.303
	3 vs. 4	Overall impression	Cohen’s kappa	0.49	0.71
		Location of parenchymal defect	Cohen’s kappa	0.43	0.63
		Percentage of kidney involvement	Kendall’s tau-b	0.43	0.64
Stage-2	1 vs. 2	Overall impression	Cohen’s kappa	0.0801	0.14
		Location of parenchymal defect	Cohen’s kappa	0.092	0.11
		Percentage of kidney involvement	Kendall’s tau-b	0.23	0.22
	1 vs. 3	Overall impression	Cohen’s kappa	0.606	0.72
		Location of parenchymal defect	Cohen’s kappa	0.49	0.64
		Percentage of kidney involvement	Kendall’s tau-b	0.49	0.63
	1 vs. 4	Overall impression	Cohen’s kappa	0.64	0.71
		Location of parenchymal defect	Cohen’s kappa	0.52	0.601
		Percentage of kidney involvement	Kendall’s tau-b	0.57	0.63
	2 vs. 3	Overall impression	Cohen’s kappa	0.092	0.13
		Location of parenchymal defect	Cohen’s kappa	0.12	0.13
		Percentage of kidney involvement	Kendall’s tau-b	0.23	0.25
	2 vs. 4	Overall impression	Cohen’s kappa	0.094	0.17
		Location of parenchymal defect	Cohen’s kappa	0.13	0.17
		Percentage of kidney involvement	Kendall’s tau-b	0.28	0.32
	3 vs. 4	Overall impression	Cohen’s kappa	0.58	0.76
		Location of parenchymal defect	Cohen’s kappa	0.51	0.68
		Percentage of kidney involvement	Kendall’s tau-b	0.48	0.68

### Annotation visualization

This procedure involved systematic analysis of multiple versions of the same medical image to identify and visualize areas of consensus between expert annotators A, B, C, and D. The goal was to ensure that annotations are consistent and reliable, which is crucial for creating high-quality datasets for training machine learning models in medical image analysis.

**Fig. 3:**
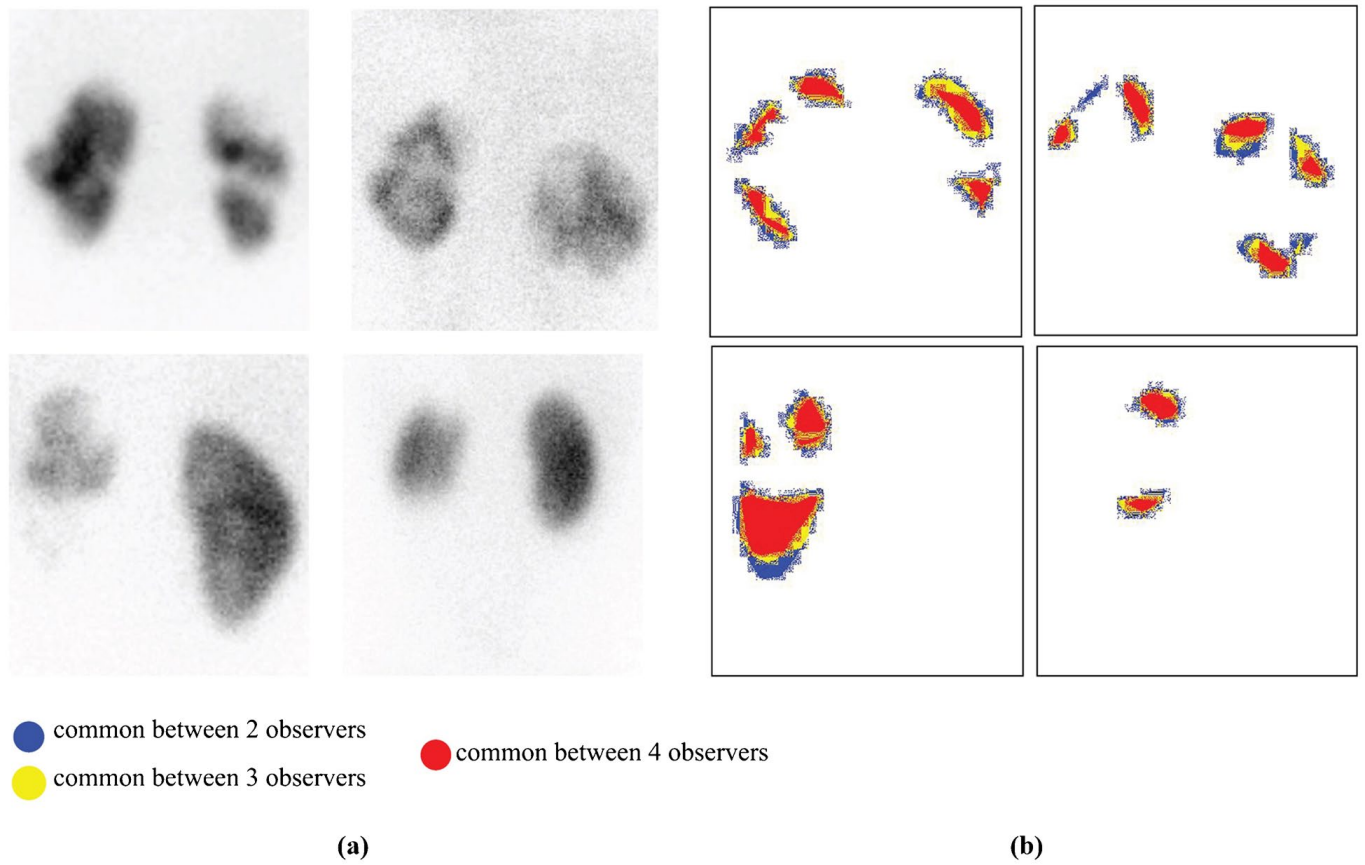
Annotation visualization. **a** Nuclear scan images. **b** Corresponding masks with inter-observer agreement overlaid (marked by two annotators, *blue pixels*; three annotators, *yellow*; all four annotators, *red pixels*)

Figure 3 demonstrates annotation visualization. For each medical image, annotations provided by the four experts were collected and aligned. Each version of the image represents a binary mask, where relevant features (e.g., regions of interest such as a specific organ or abnormality) are highlighted based on the expert’s annotations. These binary masks indicate the areas that each annotator considers significant for diagnosis. The masks were then overlaid to create a composite image that determines the number of annotators who marked each pixel as a measure of consensus. The resulting composite image is then color-coded to indicate different levels of agreement among the annotators, highlighting areas where opinions converge. Regions marked in red indicate a high level of confidence, as all experts agree on these areas, making them the most accurate and clinically relevant. Conversely, areas marked in blue, yellow, or red show varying degrees of consensus, providing insights into regions where there might be discrepancies or uncertainty. By systematically evaluating and visualizing consensus among multiple annotators, this procedure enhances dataset quality for future training of machine learning models.

## Discussion

Intra-observer agreement was consistently strong, indicating reliable individual interpretations over time. In contrast, inter-observer agreement was moderate, with notable variability in evaluating the severity of scarring and the localization of defects. These findings emphasize the challenges posed by the subjective nature of DMSA interpretation, which may impact clinical decision-making and outcomes. Addressing these issues requires greater standardization, targeted observer training, and advanced diagnostic tools to improve consistency.

DMSA scan remains the gold standard for evaluating renal cortical scarring in pediatric patients with UTIs due to its superior sensitivity and lower impact on renal function compared to ultrasound [14, 15]. Unlike ultrasound, DMSA scanning is not affected by bowel contents and does not impose osmotic burdens or alter renal function. Moreover, this method boasts a higher sensitivity in detecting acute pyelonephritis and offers comparable sensitivity to computed tomography (CT) scans, with fewer practical limitations in pediatric populations [16]. However, its diagnostic reliability still depends heavily on qualitative assessment, which introduces the potential for variability. The timing of imaging is also important, as the progression of renal parenchymal disease can affect scan interpretation.

Previous research has explored variance in DMSA scan interpretation both between and within individual observers. Erdoğan et al. [17] analyzed variation in Tc-99m renal scintigraphy using a modified grading system to rate renal parenchymal damage from 0 to V, then applied Kendall’s tau-b correlation to assess observer agreement. High agreement was found for both DMSA scans and MAG3 scans, emphasizing that clear interpretation standards and observer training can limit subjectivity. As pointed out by Erdoğan et al., variability in observer assessments is a critical factor for evaluating the reliability of diagnostic methods. This highlights the necessity for standardizing interpretation protocols to enhance consistency across observers. Mattoo et al. [18] examined DMSA scan interpretation between observers in the Randomized Intervention for Children with Vesicoureteral Reflux (RIVUR) trial. Reports from two reference radiologists were compared with reports from local radiologists who each read the scans for renal scarring and pyelonephritis. Interpretation of unusual DMSA scans was inconsistent, with the reference radiologists reporting different levels of scarring in 79% of kidneys. The reliability of scan results is crucial for guiding diagnosis, treatment, and follow-up in the clinic. In a previous study of acute pyelonephritis and renal scarring in children, Patel et al. [14] engaged two nuclear medicine physicians to separately review 57 DMSA scans for kidney shape, renal tissue anomalies, and overall impressions against specified criteria. Within and between observers, agreement rates were 95.9% and 90.6%, respectively.

However, noticeable variation between observers underscores the need for improved training and calibration among radiologists to reduce subjective differences. In our study, although intra-observer agreement was strong, inter-observer variability was evident. This inherent subjectivity is likely due to the variable experience of individual radiologists and their evaluation of complex or subtle imaging findings. It has been recognized in several studies that anatomical variations, particularly those related to age, can lead to discrepancies in interpretation. For example, variations such as pear-shaped kidneys, discrepancies in pole activity, and unusual renal column shapes can complicate the assessment process [19]. Moreover, technical challenges such as poor radiotracer uptake in neonates, as highlighted by Craig et al., can result in misinterpretations of the images [20]. In this study, some cases were challenging due to these anatomical and technical factors.

Localizing parenchymal defects and quantifying percentage of kidney involvement demonstrated moderate agreement. The lower agreement levels observed are likely due to the increased complexity and larger number of categories, unlike the binary classification of scar presence or absence. While analysis of kidney involvement demonstrated robust ordinal associations, variability persisted for intra-observer agreement and inter-observer agreement. This suggests that while observers are consistent within their own assessments, inter-observer variability is more pronounced due to differing interpretations of tissue involvement.

The analysis of inter-observer agreement revealed significant discrepancies in the consistency of kidney evaluation across different observer pairs, with noticeable variations based on both the evaluation criterion and stage of assessment. For both kidneys, the agreement for these parameters was strong, indicating a good level of consistency. These findings suggest that these observers had a shared understanding or interpretation of the scans, likely due to comparable training or experience. Several studies have noted significant variations in the interpretation of DMSA scans, with low reproducibility of cortical scintigraphy being a recurring issue [21, 22]. They advocate for the use of standardized criteria and terminology to improve the consistency of interpretation [23].

Conversely, Observer-2’s performance stood out as the main source of variability. In all pairings involving Observer-2, the agreement scores were consistently lower. This was especially evident in their evaluations of the overall impression and the location of parenchymal defects, where Cohen’s kappa values frequently fell below 0.2, highlighting poor to slight agreement. Additionally, Kendall’s tau-b coefficients in these pairs were substantially lower, indicating that Observer-2’s assessments did not align well with those of other observers. This suggests that Observer-2’s interpretation may have been influenced by factors such as different levels of expertise, varying experience, or reliance on personal judgment in the absence of strict, standardized criteria. Such subjective assessment could include variability in recognizing subtle parenchymal changes, determining the significance of focal uptake reductions, or applying inconsistent thresholds for defining renal scarring. The clinical implication of such variability is significant, as inconsistent interpretation of DMSA scans can lead to disparities in diagnosis and follow-up decisions, potentially affecting patient outcomes. In particular, underestimation or overestimation of renal scarring may influence decisions regarding long-term monitoring or interventions. This underscores the need for enhanced standardization and decision support. Future integration of artificial intelligence (AI)-based tools, trained on expertly annotated datasets, may help mitigate such discrepancies by providing consistent, reproducible assessments and flagging ambiguous cases for additional review. AI systems can serve as second readers, offering objective interpretation benchmarks and assisting in harmonizing diagnostic judgments across observers with varying levels of experience.

Higher agreement levels were observed in parameters where standardized criteria and clear diagnostic guidelines were more effectively applied. Indeed, the benefits of employing a consistent approach to scan interpretation were illustrated by the high intra-observer agreement for Observer-4, who achieved kappa scores of 0.91 (left) and 0.95 (right) for overall impressions. This consistency is most likely the result of comprehensive training, strict adherence to standardized protocols, and a greater amount of experience in interpreting DMSA scans. Additionally, agreement tends to be higher in more straightforward cases with clear indications of renal scarring, as these are less prone to subjective variation. These data also underscore the value of using robust statistical validation to measure consistency in medical imaging studies.

While this study offers valuable insights, there remain several avenues for future improvement. The sample was relatively small and drawn from a single center, which may limit the broader applicability of the findings. Future studies involving larger, multicenter cohorts would help enhance generalizability. Additionally, although the retrospective design allowed efficient data collection and review, prospective studies could offer more control over potential biases and enable standardized imaging and reporting protocols from the outset. The current analysis relied solely on posterior planar DMSA images obtained with a parallel-hole collimator. While widely used in clinical practice, planar imaging does not provide the spatial resolution or three-dimensional detail achievable with single-photon emission computed tomography (SPECT) or pinhole collimator images. Incorporating such advanced imaging modalities in future research could improve the detection of subtle cortical abnormalities and help reduce inter-observer variability. These enhancements may ultimately contribute to more robust and accurate interpretation frameworks for pediatric renal imaging.

## Conclusion

This study demonstrates that while intra-observer agreement in technetium-99m dimercaptosuccinic acid scan interpretation is strong, inter-observer variability remains a challenge, particularly in assessing renal scarring and parenchymal defects. These findings underscore the need for standardized interpretation protocols, targeted observer training, and the potential integration of advanced imaging modalities to improve diagnostic consistency and support more accurate clinical decision-making in pediatric patients.

## Supplementary Information

Below is the link to the electronic supplementary material.

Supplementary file 1 (DOCX 8.30 MB)

## Data Availability

No datasets were generated or analysed during the current study.
