# Supplementary material for: How consistent is the interpretation of renal scarring in pediatric patients using technetium-99m dimercaptosuccinic acid scintigraphy
Source: Pediatr Radiol. 2025 Aug 26;55(12):2632–42. doi: 10.1007/s00247-025-06379-z (PMC12602622; doi:10.1007/s00247-025-06379-z)
Supplement: Supplementary file 1 — (DOCX 8.30 MB) [file 247_2025_6379_MOESM1_ESM.docx]

**Supplementary materials**


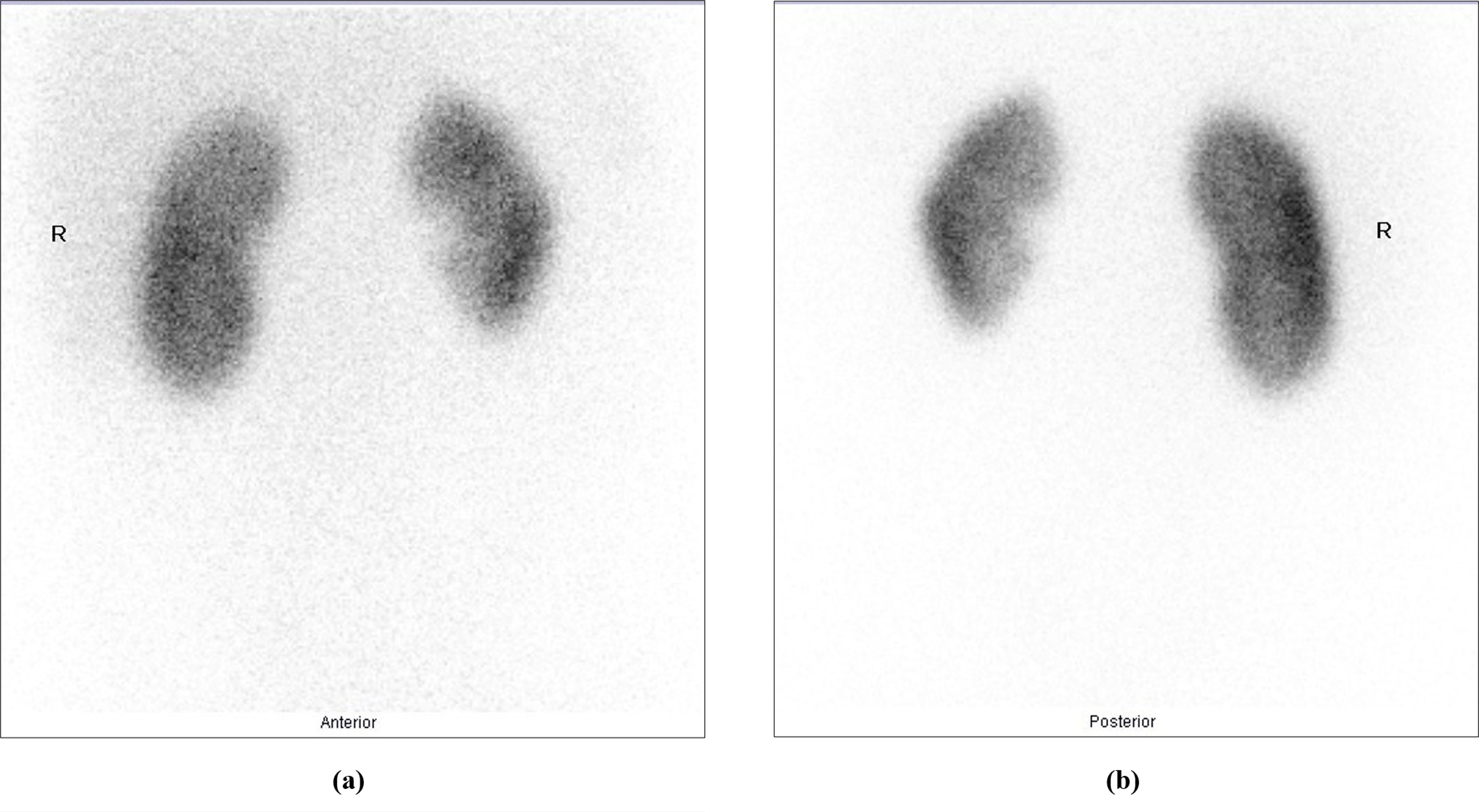


**Supplementary material 1.** Tc-99m dimercaptosuccinic acid scan, **a.** anterior static picture, **b.** posterior static picture


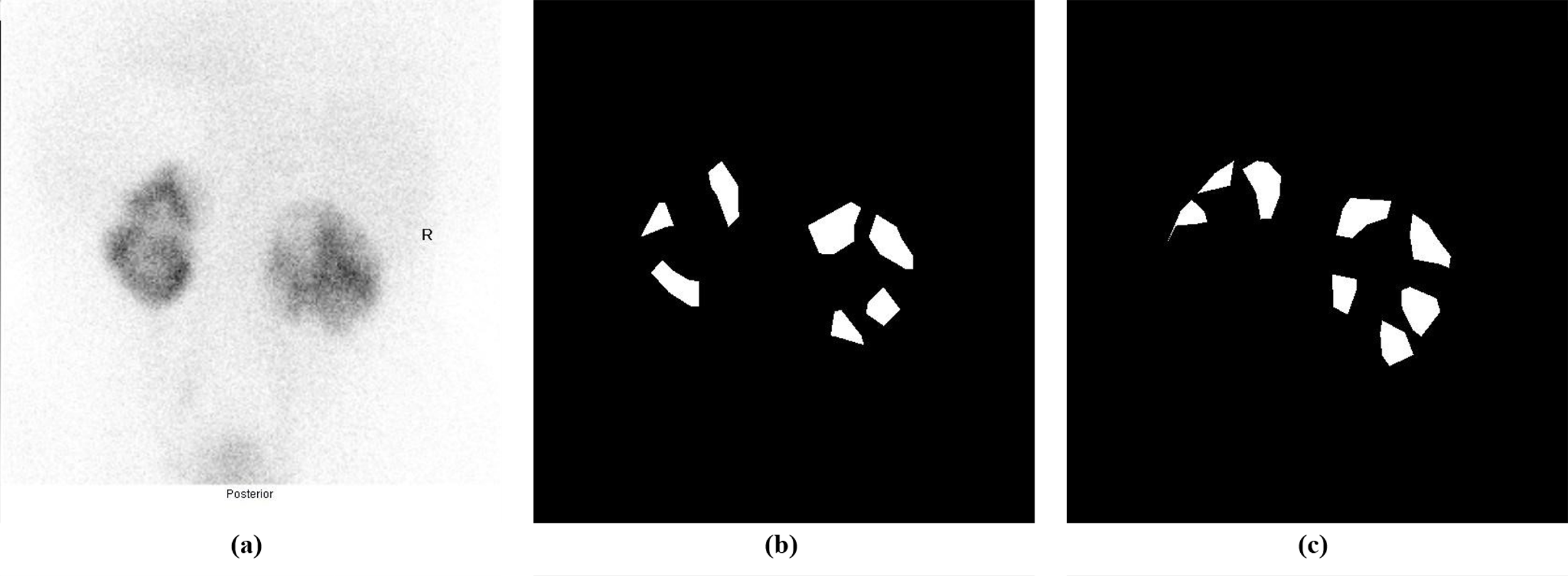


**Supplementary material 2.** Annotation mask of kidney scar. **a.** Tc-99m dimercaptosuccinic acid scan, **b.** first stage annotation, **c.** second stage annotation

**Intra-observer agreement:**

Supplementary material 3. Intra-observer agreement by observer-1

| **Overall impression of left and right kidney** | |
| --- | --- |
| \| **Left Kidney** \| Stage - 2 \| \| \| \| \| --- \| --- \| --- \| --- \| --- \| \| Stage - 1 \|  \| No Scar \| Scar \| All \| \| No Scar \| 143 \| 5 \| 148 \| \| Scar \| 22 \| 50 \| 72 \| \| All \| 165 \| 55 \| 220 \| \| Cohen's Kappa Score: \| 0.703 \| \| \| \| | \| **Right Kidney** \| Stage - 2 \| \| \| \| \| --- \| --- \| --- \| --- \| --- \| \| Stage - 1 \|  \| No Scar \| Scar \| All \| \| No Scar \| 150 \| 4 \| 154 \| \| Scar \| 16 \| 50 \| 66 \| \| All \| 166 \| 54 \| 220 \| \| Cohen's Kappa Score: \| 0.77 \| \| \| \| |
| **Location of parenchymal defect of left and right Kidney** | |
| \| **Left Kidney** \| Stage - 2 \| \| \| \| \| \| \| \| --- \| --- \| --- \| --- \| --- \| --- \| --- \| --- \| \| Stage - 1 \|  \| Not Affected \| Upper pole \| Mid-zone \| Lower pole \| Multiple Zone \| All \| \| Not Affected \| 144 \| 2 \| 1 \| 1 \| 0 \| 148 \| \| Upper pole \| 7 \| 11 \| 1 \| 0 \| 0 \| 19 \| \| Mid-zone \| 0 \| 0 \| 1 \| 0 \| 0 \| 1 \| \| Lower pole \| 8 \| 2 \| 1 \| 4 \| 1 \| 16 \| \| Multiple Zone \| 6 \| 7 \| 1 \| 3 \| 19 \| 36 \| \| All \| 165 \| 22 \| 5 \| 8 \| 20 \| 220 \| \| Cohen's Kappa Score \| 0.602 \| \| \| \| \| \| \| | \| **Right Kidney** \| Stage - 2 \| \| \| \| \| \| \| \| --- \| --- \| --- \| --- \| --- \| --- \| --- \| --- \| \| Stage - 1 \|  \| Not Affected \| Upper pole \| Mid-zone \| Lower pole \| Multiple Zone \| All \| \| Not Affected \| 151 \| 0 \| 0 \| 4 \| 0 \| 155 \| \| Upper pole \| 3 \| 11 \| 1 \| 0 \| 3 \| 18 \| \| Mid-zone \| 0 \| 0 \| 2 \| 0 \| 0 \| 2 \| \| Lower pole \| 5 \| 2 \| 0 \| 9 \| 3 \| 19 \| \| Multiple Zone \| 7 \| 5 \| 0 \| 2 \| 12 \| 26 \| \| All \| 166 \| 18 \| 3 \| 15 \| 18 \| 220 \| \| Cohen's Kappa Score \| 0.64 \| \| \| \| \| \| \| |
| **Percentage of kidney involvement of left and right kidney** | |
| \| **Left Kidney** \| Stage 2 \| \| \| \| \| \| \| \| \| --- \| --- \| --- \| --- \| --- \| --- \| --- \| --- \| --- \| \| Stage 1 \|  \| 0% \| <10% \| 10-24% \| 25-49% \| 50-74% \| >74% \| All \| \| 0% \| 143 \| 5 \| 0 \| 0 \| 0 \| 0 \| 148 \| \| <10% \| 14 \| 14 \| 4 \| 0 \| 0 \| 0 \| 32 \| \| 10-24% \| 3 \| 6 \| 12 \| 1 \| 0 \| 0 \| 22 \| \| 25-49% \| 2 \| 4 \| 1 \| 1 \| 0 \| 0 \| 8 \| \| 50-74% \| 3 \| 0 \| 1 \| 2 \| 1 \| 0 \| 7 \| \| >74% \| 0 \| 1 \| 0 \| 1 \| 1 \| 0 \| 3 \| \| All \| 165 \| 30 \| 18 \| 5 \| 2 \| 0 \| 220 \| \| Kendall's Tau-b \| 0.62 \| \| \| \| \| \| \| \| | \| **Right Kidney** \| Stage 2 \| \| \| \| \| \| \| \| \| --- \| --- \| --- \| --- \| --- \| --- \| --- \| --- \| --- \| \| Stage 1 \|  \| 0% \| <10% \| 10-24% \| 25-49% \| 50-74% \| >74% \| All \| \| 0% \| 148 \| 4 \| 0 \| 0 \| 0 \| 0 \| 152 \| \| <10% \| 8 \| 19 \| 4 \| 0 \| 0 \| 0 \| 31 \| \| 10-24% \| 6 \| 7 \| 8 \| 1 \| 0 \| 0 \| 22 \| \| 25-49% \| 2 \| 1 \| 3 \| 3 \| 0 \| 0 \| 9 \| \| 50-74% \| 2 \| 0 \| 2 \| 1 \| 0 \| 0 \| 5 \| \| >74% \| 0 \| 0 \| 0 \| 1 \| 0 \| 0 \| 1 \| \| All \| 166 \| 31 \| 17 \| 6 \| 0 \| 0 \| 220 \| \| Kendall's Tau-b \| 0.69 \| \| \| \| \| \| \| \| |


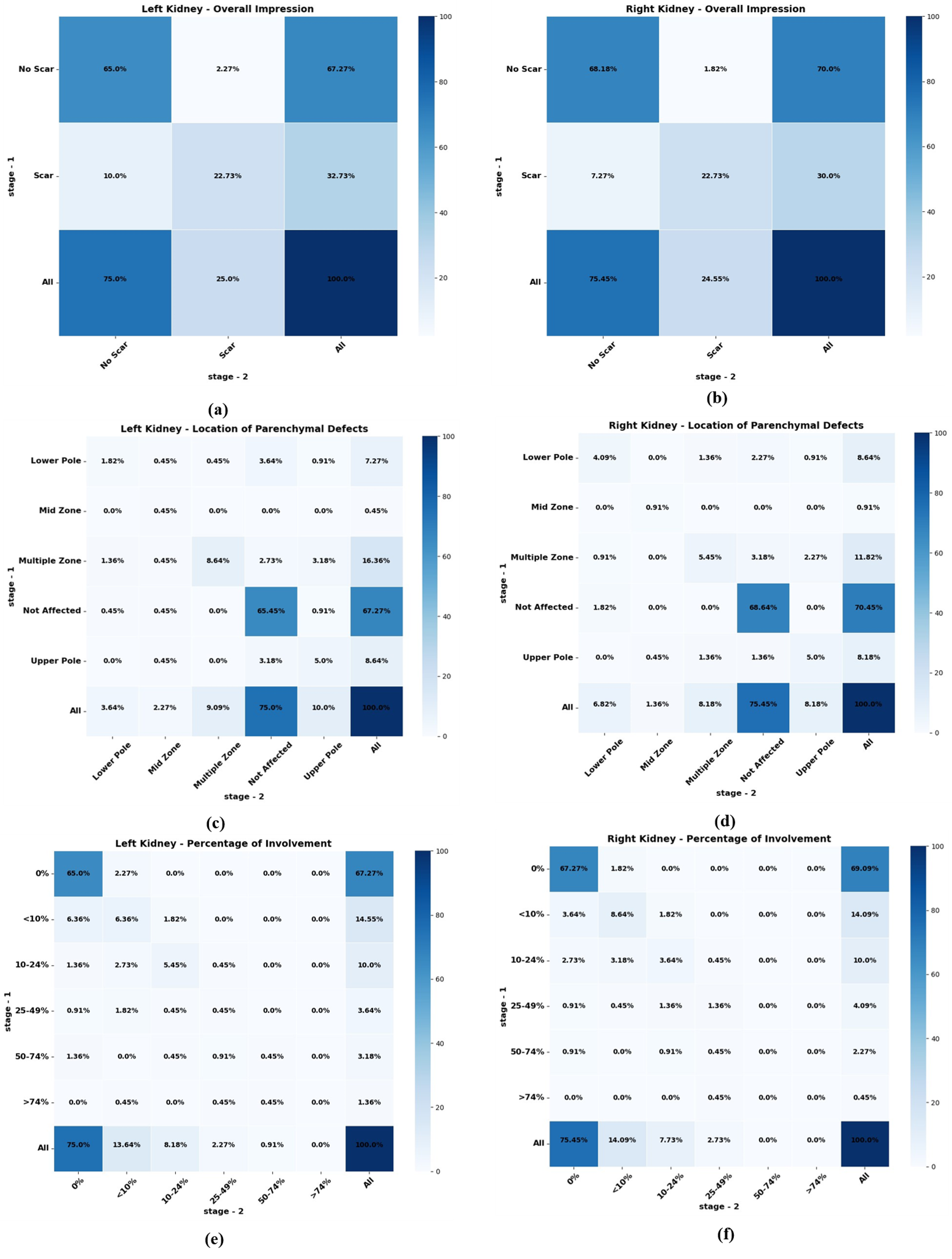


**Supplementary material 4.** Heatmap of intra-observer agreement by observer-1. Cohen's Kappa cross-tabulations for overall impression **a.** left kidney, **b.** right kidney. Cohen's Kappa cross-tabulations for location of parenchymal defect **c.** left kidney, **d.** right kidney. Kendall's Tau-b cross-tabulations for percent involvement of **e.** left kidney, **f.** right kidney

**Supplementary material 5.** Intra-observer agreement by observer-2.

| **Overall impression of left and right kidney** | |
| --- | --- |
| \| **Left Kidney** \| Stage - 2 \| \| \| \| \| --- \| --- \| --- \| --- \| --- \| \| Stage - 1 \|  \| No Scar \| Scar \| All \| \| No Scar \| 20 \| 10 \| 30 \| \| Scar \| 4 \| 184 \| 188 \| \| All \| 24 \| 194 \| 218 \| \| Cohen's Kappa Score: \| 0.704 \| \| \| \| | \| **Right Kidney** \| Stage - 2 \| \| \| \| \| --- \| --- \| --- \| --- \| --- \| \| Stage - 1 \|  \| No Scar \| Scar \| All \| \| No Scar \| 38 \| 15 \| 53 \| \| Scar \| 4 \| 161 \| 165 \| \| All \| 42 \| 176 \| 218 \| \| Cohen's Kappa Score: \| 0.74 \| \| \| \| |
| **Location of parenchymal defect of left and right kidney** | |
| \| **Left Kidney** \| Stage - 2 \| \| \| \| \| \| \| \| --- \| --- \| --- \| --- \| --- \| --- \| --- \| --- \| \| Stage - 1 \|  \| Not Affected \| Upper pole \| Mid-zone \| Lower pole \| Multiple Zone \| All \| \| Not Affected \| 18 \| 5 \| 2 \| 1 \| 4 \| 30 \| \| Upper pole \| 2 \| 36 \| 4 \| 0 \| 31 \| 73 \| \| Mid-zone \| 0 \| 0 \| 7 \| 1 \| 4 \| 12 \| \| Lower pole \| 1 \| 1 \| 0 \| 1 \| 4 \| 7 \| \| Multiple Zone \| 0 \| 6 \| 2 \| 2 \| 86 \| 96 \| \| All \| 21 \| 48 \| 15 \| 5 \| 129 \| 218 \| \| Cohen's Kappa Score \| 0.504 \| \| \| \| \| \| \| | \| **Right Kidney** \| Stage - 2 \| \| \| \| \| \| \| \| --- \| --- \| --- \| --- \| --- \| --- \| --- \| --- \| \| Stage - 1 \|  \| Not Affected \| Upper pole \| Mid-zone \| Lower pole \| Multiple Zone \| All \| \| Not Affected \| 38 \| 5 \| 1 \| 0 \| 9 \| 53 \| \| Upper pole \| 3 \| 40 \| 2 \| 0 \| 34 \| 79 \| \| Mid-zone \| 0 \| 0 \| 3 \| 0 \| 3 \| 6 \| \| Lower pole \| 0 \| 0 \| 0 \| 2 \| 2 \| 4 \| \| Multiple Zone \| 1 \| 4 \| 3 \| 0 \| 68 \| 76 \| \| All \| 42 \| 49 \| 9 \| 2 \| 116 \| 218 \| \| Cohen's Kappa Score \| 0.55 \| \| \| \| \| \| \| |
| **Percentage of kidney involvement of left and right kidney** | |
| \| **Left Kidney** \| Stage 2 \| \| \| \| \| \| \| \| \| --- \| --- \| --- \| --- \| --- \| --- \| --- \| --- \| --- \| \| Stage 1 \|  \| 0% \| <10% \| 10-24% \| 25-49% \| 50-74% \| >74% \| All \| \| 0% \| 20 \| 7 \| 2 \| 0 \| 1 \| 0 \| 30 \| \| <10% \| 1 \| 14 \| 17 \| 4 \| 1 \| 0 \| 37 \| \| 10-24% \| 2 \| 16 \| 41 \| 9 \| 6 \| 2 \| 76 \| \| 25-49% \| 1 \| 5 \| 14 \| 4 \| 5 \| 2 \| 31 \| \| 50-74% \| 0 \| 1 \| 1 \| 3 \| 10 \| 4 \| 19 \| \| >74% \| 0 \| 0 \| 0 \| 0 \| 2 \| 23 \| 25 \| \| All \| 24 \| 43 \| 75 \| 20 \| 25 \| 31 \| 218 \| \| Kendall's Tau-b \| 0.43 \| \| \| \| \| \| \| \| | \| **Right Kidney** \| Stage 2 \| \| \| \| \| \| \| \| \| --- \| --- \| --- \| --- \| --- \| --- \| --- \| --- \| --- \| \| Stage 1 \|  \| 0% \| <10% \| 10-24% \| 25-49% \| 50-74% \| >74% \| All \| \| 0% \| 38 \| 6 \| 7 \| 1 \| 0 \| 1 \| 53 \| \| <10% \| 2 \| 17 \| 12 \| 1 \| 2 \| 0 \| 34 \| \| 10-24% \| 2 \| 17 \| 29 \| 16 \| 1 \| 1 \| 66 \| \| 25-49% \| 1 \| 0 \| 7 \| 7 \| 12 \| 2 \| 29 \| \| 50-74% \| 0 \| 0 \| 1 \| 4 \| 7 \| 1 \| 13 \| \| >74% \| 0 \| 0 \| 1 \| 0 \| 3 \| 19 \| 23 \| \| All \| 43 \| 40 \| 57 \| 29 \| 25 \| 24 \| 218 \| \| Kendall's Tau-b \| 0.52 \| \| \| \| \| \| \| \| |


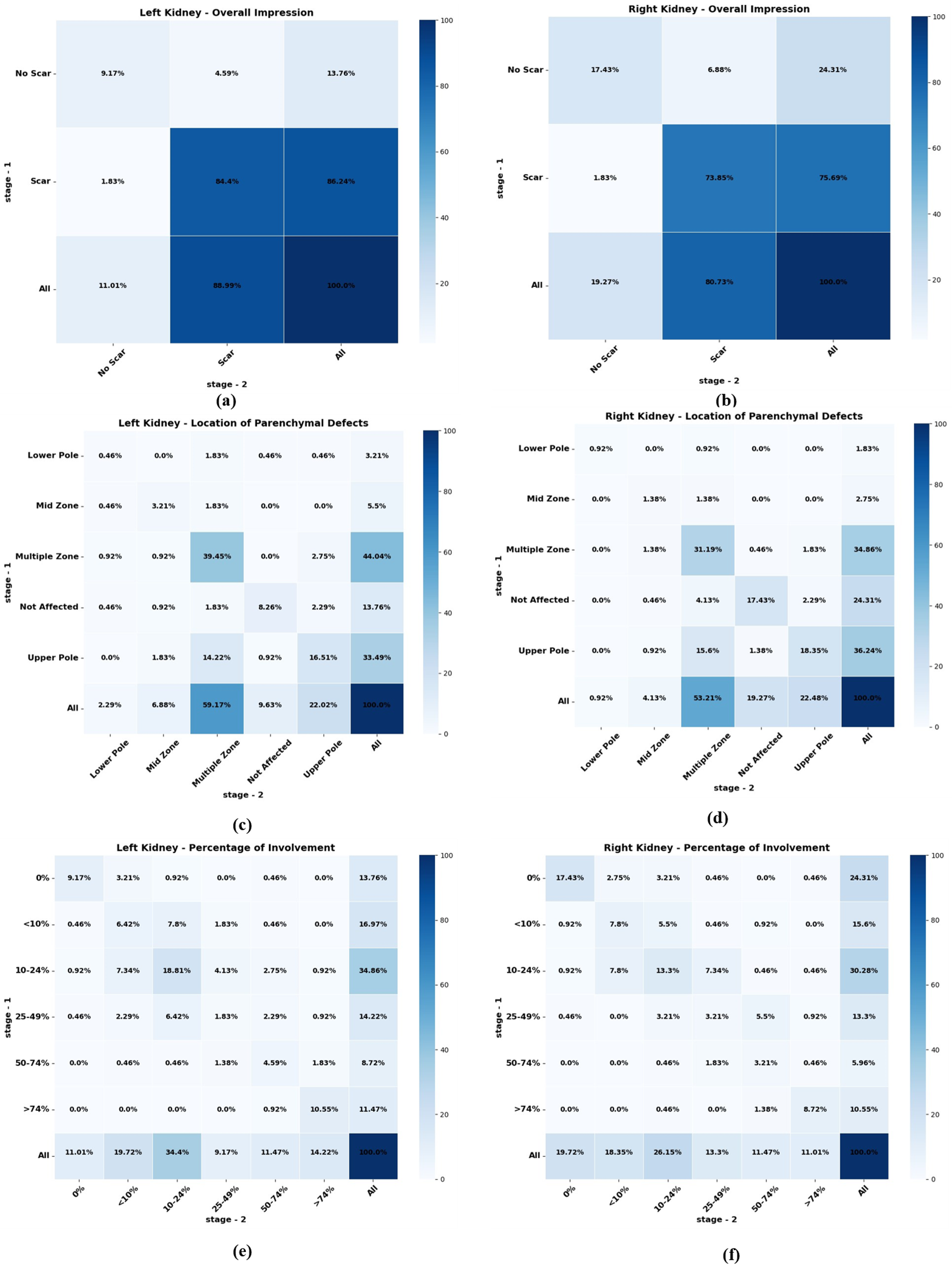


**Supplementary material 6.** Heatmap of intra-observer agreement by observer-2. Cohen's Kappa cross-tabulations for overall impression **a.** left kidney, **b.** right kidney. Cohen's Kappa cross-tabulations for location of parenchymal defect **c.** left kidney, **d.** right kidney. Kendall's Tau-b cross-tabulations for percent involvement of **e.** left kidney, **f.** right kidney

**Supplementary material 7.** Intra-observer agreement by observer-3

| **Overall impression of left and right kidney** | |
| --- | --- |
| \| **Left Kidney** \| Stage - 2 \| \| \| \| \| --- \| --- \| --- \| --- \| --- \| \| Stage - 1 \|  \| No Scar \| Scar \| All \| \| No Scar \| 141 \| 5 \| 146 \| \| Scar \| 11 \| 63 \| 74 \| \| All \| 152 \| 68 \| 220 \| \| Cohen's Kappa Score: \| 0.83 \| \| \| \| | \| **Right Kidney** \| Stage - 2 \| \| \| \| \| --- \| --- \| --- \| --- \| --- \| \| Stage - 1 \|  \| No Scar \| Scar \| All \| \| No Scar \| 163 \| 4 \| 167 \| \| Scar \| 7 \| 46 \| 53 \| \| All \| 170 \| 50 \| 220 \| \| Cohen's Kappa Score: \| 0.86 \| \| \| \| |
| **Location of parenchymal defect of left and right kidney** | |
| \| **Left Kidney** \| Stage - 2 \| \| \| \| \| \| \| \| --- \| --- \| --- \| --- \| --- \| --- \| --- \| --- \| \| Stage - 1 \|  \| Not Affected \| Upper pole \| Mid-zone \| Lower pole \| Multiple Zone \| All \| \| Not Affected \| 139 \| 3 \| 2 \| 0 \| 0 \| 144 \| \| Upper pole \| 3 \| 10 \| 0 \| 1 \| 2 \| 16 \| \| Mid-zone \| 2 \| 1 \| 5 \| 0 \| 3 \| 11 \| \| Lower pole \| 4 \| 0 \| 0 \| 7 \| 2 \| 13 \| \| Multiple Zone \| 2 \| 0 \| 2 \| 3 \| 29 \| 36 \| \| All \| 150 \| 14 \| 9 \| 11 \| 36 \| 220 \| \| Cohen's Kappa Score \| 0.73 \| \| \| \| \| \| \| | \| **Right Kidney** \| Stage - 2 \| \| \| \| \| \| \| \| --- \| --- \| --- \| --- \| --- \| --- \| --- \| --- \| \| Stage - 1 \|  \| Not Affected \| Upper pole \| Mid-zone \| Lower pole \| Multiple Zone \| All \| \| Not Affected \| 163 \| 0 \| 1 \| 2 \| 1 \| 167 \| \| Upper pole \| 1 \| 12 \| 0 \| 1 \| 1 \| 15 \| \| Mid-zone \| 1 \| 0 \| 3 \| 0 \| 0 \| 4 \| \| Lower pole \| 4 \| 1 \| 0 \| 5 \| 1 \| 11 \| \| Multiple Zone \| 1 \| 0 \| 0 \| 0 \| 22 \| 23 \| \| All \| 170 \| 13 \| 4 \| 8 \| 25 \| 220 \| \| Cohen's Kappa Score \| 0.82 \| \| \| \| \| \| \| |
| **Percentage of kidney involvement of left and right kidney** | |
| \| **Left Kidney** \| Stage 2 \| \| \| \| \| \| \| \| \| --- \| --- \| --- \| --- \| --- \| --- \| --- \| --- \| --- \| \| Stage 1 \|  \| 0% \| <10% \| 10-24% \| 25-49% \| 50-74% \| >74% \| All \| \| 0% \| 145 \| 5 \| 0 \| 0 \| 0 \| 0 \| 150 \| \| <10% \| 8 \| 11 \| 6 \| 1 \| 0 \| 0 \| 26 \| \| 10-24% \| 1 \| 4 \| 13 \| 8 \| 1 \| 0 \| 27 \| \| 25-49% \| 0 \| 0 \| 1 \| 10 \| 2 \| 0 \| 13 \| \| 50-74% \| 0 \| 0 \| 0 \| 0 \| 3 \| 0 \| 3 \| \| >74% \| 0 \| 0 \| 0 \| 0 \| 0 \| 1 \| 1 \| \| All \| 154 \| 20 \| 20 \| 19 \| 6 \| 1 \| 220 \| \| Kendall's Tau-b \| 0.73 \| \| \| \| \| \| \| \| | \| **Right Kidney** \| Stage 2 \| \| \| \| \| \| \| \| \| --- \| --- \| --- \| --- \| --- \| --- \| --- \| --- \| --- \| \| Stage 1 \|  \| 0% \| <10% \| 10-24% \| 25-49% \| 50-74% \| >74% \| All \| \| 0% \| 162 \| 3 \| 2 \| 1 \| 0 \| 0 \| 168 \| \| <10% \| 3 \| 11 \| 8 \| 0 \| 0 \| 0 \| 22 \| \| 10-24% \| 4 \| 0 \| 10 \| 4 \| 0 \| 0 \| 18 \| \| 25-49% \| 0 \| 0 \| 0 \| 8 \| 3 \| 0 \| 11 \| \| 50-74% \| 0 \| 0 \| 0 \| 0 \| 1 \| 0 \| 1 \| \| >74% \| 0 \| 0 \| 0 \| 0 \| 0 \| 0 \| 0 \| \| All \| 169 \| 14 \| 20 \| 13 \| 4 \| 0 \| 220 \| \| Kendall's Tau-b \| 0.78 \| \| \| \| \| \| \| \| |

**Supplementary material 8.** Intra-observer agreement by observer-4

| **Overall impression of left and right kidney** | |
| --- | --- |
| \| **Left Kidney** \| Stage - 2 \| \| \| \| \| --- \| --- \| --- \| --- \| --- \| \| Stage - 1 \|  \| No Scar \| Scar \| All \| \| No Scar \| 136 \| 1 \| 137 \| \| Scar \| 8 \| 75 \| 83 \| \| All \| 144 \| 76 \| 220 \| \| Cohen's Kappa Score: \| 0.91 \| \| \| \| | \| **Right Kidney** \| Stage - 2 \| \| \| \| \| --- \| --- \| --- \| --- \| --- \| \| Stage - 1 \|  \| No Scar \| Scar \| All \| \| No Scar \| 154 \| 0 \| 154 \| \| Scar \| 4 \| 62 \| 66 \| \| All \| 158 \| 62 \| 220 \| \| Cohen's Kappa Score: \| 0.95 \| \| \| \| |
| **Location of parenchymal defect of left and right kidney** | |
| \| **Left Kidney** \| Stage - 2 \| \| \| \| \| \| \| \| --- \| --- \| --- \| --- \| --- \| --- \| --- \| --- \| \| Stage - 1 \|  \| Not Affected \| Upper pole \| Mid-zone \| Lower pole \| Multiple Zone \| All \| \| Not Affected \| 136 \| 0 \| 0 \| 1 \| 0 \| 137 \| \| Upper pole \| 3 \| 13 \| 0 \| 0 \| 0 \| 16 \| \| Mid-zone \| 1 \| 0 \| 6 \| 0 \| 0 \| 7 \| \| Lower pole \| 1 \| 0 \| 0 \| 11 \| 1 \| 13 \| \| Multiple Zone \| 4 \| 1 \| 0 \| 0 \| 42 \| 47 \| \| All \| 145 \| 14 \| 6 \| 12 \| 43 \| 220 \| \| Cohen's Kappa Score \| 0.89 \| \| \| \| \| \| \| | \| **Right Kidney** \| Stage - 2 \| \| \| \| \| \| \| \| --- \| --- \| --- \| --- \| --- \| --- \| --- \| --- \| \| Stage - 1 \|  \| Not Affected \| Upper pole \| Mid-zone \| Lower pole \| Multiple Zone \| All \| \| Not Affected \| 155 \| 1 \| 0 \| 0 \| 0 \| 156 \| \| Upper pole \| 1 \| 9 \| 0 \| 0 \| 0 \| 10 \| \| Mid-zone \| 1 \| 0 \| 2 \| 0 \| 0 \| 3 \| \| Lower pole \| 1 \| 0 \| 0 \| 11 \| 0 \| 12 \| \| Multiple Zone \| 1 \| 1 \| 1 \| 2 \| 34 \| 39 \| \| All \| 159 \| 11 \| 3 \| 13 \| 34 \| 220 \| \| Cohen's Kappa Score \| 0.909 \| \| \| \| \| \| \| |
| **Percentage of kidney involvement of left and right kidney** | |
| \| **Left Kidney** \| Stage 2 \| \| \| \| \| \| \| \| \| --- \| --- \| --- \| --- \| --- \| --- \| --- \| --- \| --- \| \| Stage 1 \|  \| 0% \| <10% \| 10-24% \| 25-49% \| 50-74% \| >74% \| All \| \| 0% \| 139 \| 0 \| 0 \| 1 \| 0 \| 1 \| 141 \| \| <10% \| 3 \| 21 \| 4 \| 0 \| 0 \| 0 \| 28 \| \| 10-24% \| 3 \| 5 \| 11 \| 2 \| 0 \| 0 \| 21 \| \| 25-49% \| 0 \| 0 \| 7 \| 1 \| 0 \| 0 \| 8 \| \| 50-74% \| 1 \| 0 \| 1 \| 1 \| 4 \| 0 \| 7 \| \| >74% \| 0 \| 0 \| 0 \| 0 \| 1 \| 14 \| 15 \| \| All \| 146 \| 26 \| 23 \| 5 \| 5 \| 15 \| 220 \| \| Kendall's Tau-b: \| 0.86 \| \| \| \| \| \| \| \| | \| **Right Kidney** \| Stage 2 \| \| \| \| \| \| \| \| \| --- \| --- \| --- \| --- \| --- \| --- \| --- \| --- \| --- \| \| Stage 1 \|  \| 0% \| <10% \| 10-24% \| 25-49% \| 50-74% \| >74% \| All \| \| 0% \| 155 \| 0 \| 0 \| 0 \| 0 \| 0 \| 155 \| \| <10% \| 1 \| 20 \| 7 \| 0 \| 0 \| 0 \| 28 \| \| 10-24% \| 0 \| 2 \| 10 \| 2 \| 0 \| 0 \| 14 \| \| 25-49% \| 1 \| 0 \| 2 \| 1 \| 3 \| 0 \| 7 \| \| 50-74% \| 1 \| 0 \| 0 \| 2 \| 6 \| 1 \| 10 \| \| >74% \| 0 \| 0 \| 0 \| 0 \| 1 \| 5 \| 6 \| \| All \| 158 \| 22 \| 19 \| 5 \| 10 \| 6 \| 220 \| \| Kendall's Tau-b: \| 0.91 \| \| \| \| \| \| \| \| |


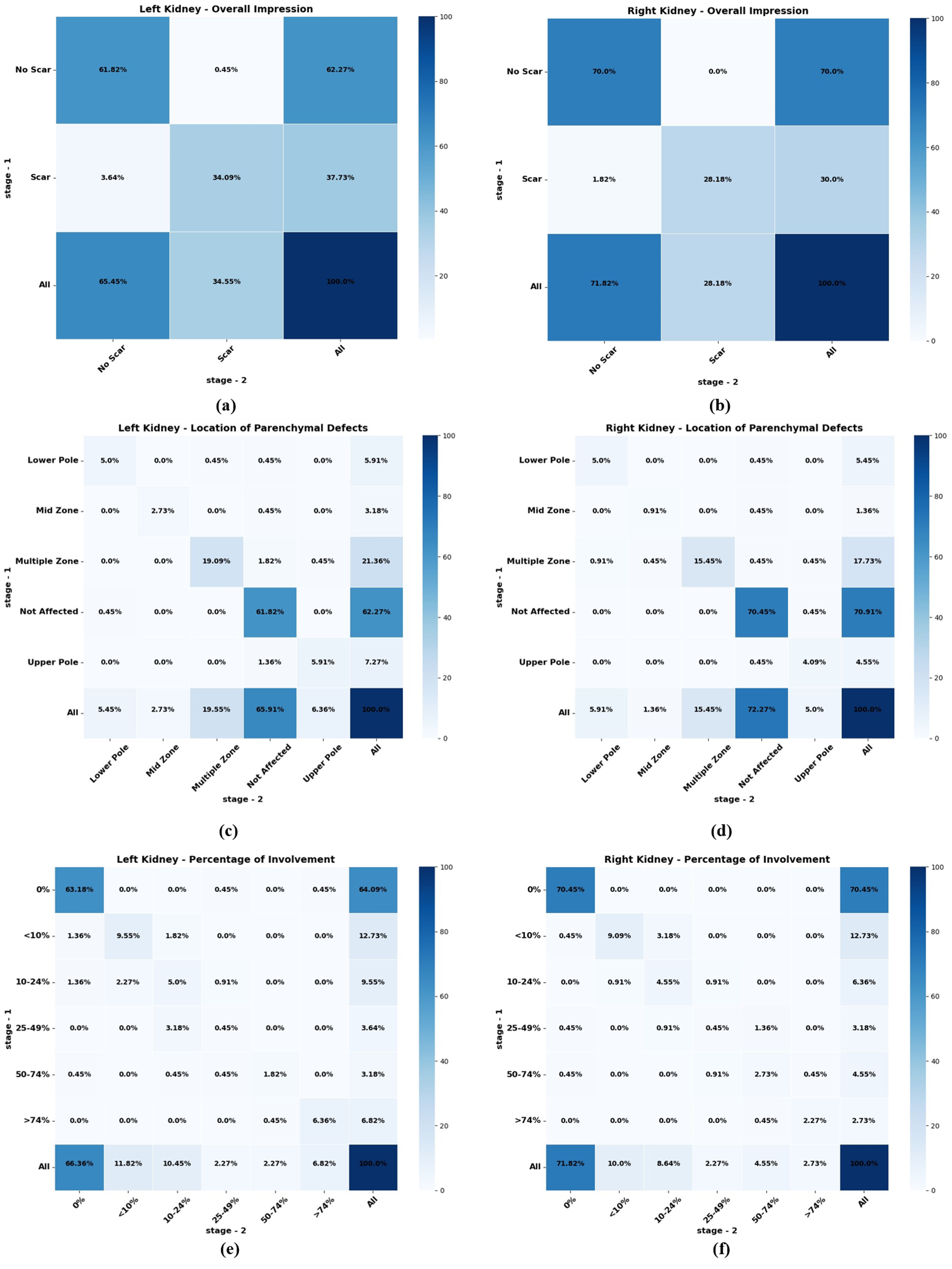


**Supplementary material 9.** Heatmap of intra-observer agreement by observer-4. Cohen's Kappa cross-tabulations for overall impression **a.** left kidney, **b.** right kidney. Cohen's Kappa cross-tabulations for location of parenchymal defect **c.** left kidney, **d.** right kidney. Kendall's Tau-b cross-tabulations for percent involvement of **e.** left kidney, **f.** right kidney
